# Improving Access to Psilocybin-Assisted Therapy: Barriers, Challenges, and Recommendations

**DOI:** 10.1192/j.eurpsy.2024.1252

**Published:** 2024-08-27

**Authors:** V. Tsang, C. Roney

**Affiliations:** UBC, Vancouver, Canada

## Abstract

**Introduction:**

Psilocybin-assisted therapy (PAT) has demonstrated significant potential in alleviating anxiety, depression, and psychological distress among individuals with terminal illnesses. However, numerous barriers prevent equitable access to this transformative treatment.

**Objectives:**

This study seeks to gather the perspectives of patients on the waitlist of PAT.

**Methods:**

Semi-structured interviews highlight the challenges faced by patients seeking PAT and their care providers and propose recommendations to enhance accessibility.

**Results:**

Through a case study of Roots to Thrive, a non-profit healthcare practice offering group-based PAT, obstacles such as complex application processes, fear of judgement, logistical and financial constraints, and systemic inequities are revealed. Moreover, Health Canada’s stringent control of PAT access via clinical trials and the Special Access Program (SAP) presents challenges for primary care providers and hinders the involvement of trained practitioners. The moral distress experienced by patients and providers due to delayed or denied access further emphasizes the urgency of addressing these barriers.

**Conclusions:**

Advocates are calling for streamlined referral systems, expedited services for end-of-life patients, formal billing infrastructure, practitioner education, expanded coverage, legislative adjustments, post-therapy support, and collaboration with non-profit organizations and Indigenous Healers to promote equitable and effective PAT. By implementing these recommendations, barriers to PAT can be overcome, allowing more individuals to benefit from this therapy and find relief from the psychological distress associated with their conditions.

**Disclosure of Interest:**

None Declared

